# Based on network pharmacology, gastrodin attenuates hypertension-induced vascular smooth muscle cell proliferation and PI3K/AKT pathway activation

**DOI:** 10.1038/s41598-023-39202-6

**Published:** 2023-07-26

**Authors:** Aling Shen, Meizhu Wu, Farman Ali, Zhi Guo, Yi Fang, Yuting Zhou, Siyu Zhang, Wenqiang Zhang, Ying Wen, Min Yu, Jun Peng, Keji Chen

**Affiliations:** 1 Postdoctoral Workstation, Department of Research and development, Tianjiang Pharmaceutical Co., Ltd., No.1 Xin Sheng Road, Jiangyin, 214400 Jiangsu China; 2 Department of Cardiology, Xiyuan Hospital of China Academy of Chinese Medical Sciences, 1 XiyuanCaochang, Hai Dian District, Beijing, 100091 China; 3 National Clinical Research Center for Cardiovascular Diseases of Traditional Chinese Medicine, Beijing, 100091 China; 4 Clinical Research Institute, The Second Affiliated Hospital & Academy of Integrative Medicine, Fujian University of Traditional Chinese Medicine, 1 Qiuyang Road, MinhouShangjie, Fuzhou, 350122 Fujian China; 5 Fujian Key Laboratory of Integrative Medicine on Geriatrics, Fujian University of Traditional Chinese Medicine, Fuzhou, 350122 Fujian China; 6 Fujian Collaborative Innovation Center for Integrative Medicine in Prevention and Treatment of Major Chronic Cardiovascular Diseases, Fuzhou, 350122 Fujian China

**Keywords:** Molecular biology, Plant sciences, Medical research

## Abstract

The effects and underlying mechanisms of gastrodin treatment on hypertensive vascular dysfunction and proliferation of vascular smooth muscle cells (VSMCs) were determined in vitro and in vivo. Using a pharmacological target network interaction analysis, 151 common targets and a PPI network were identified containing the top 10 hub genes. Kyoto encyclopedia of genes and genomes (KEGG) analysis identified the PI3K/AKT pathway as a significantly enriched pathway. Both spontaneous hypertensive rats (SHRs) and Wistar Kyoto rats were used to assess the therapeutic effects of gastrodin on hypertension. Gastrodin treatment of the SHRs resulted in a marked attenuation of elevated blood pressure, pulse wave velocity, and pathological changes in the abdominal aorta. Moreover, gastrodin treatment significantly inhibited cell growth and downregulated the expression of PCNA as well as the p-PI3K/PI3K and p-AKT/AKT levels in angiotensin II-stimulated VSMCs. Taken together**,** gastrodin treatment attenuates blood pressure elevation, vascular dysfunction, and proliferation of VSMCs and inhibits the activation of the PI3K/AKT pathway.

## Introduction

Hypertension is a major risk factor for heart disease and usually leads to vascular remodeling, cardiac failure, end-stage kidney disease, myocardial infarction, stroke, and other potentially fatal illnesses^1,2^. Therefore, controlling hypertension with medicines or compounds is an essential strategy to prevent disease onset and progression. Although awareness, the continued development of therapies, and use of combined antihypertensive medications has increased, an effective treatment rate for hypertension remains low^3,4^. Thus, there is an immediate need to identify and develop novel alternative therapeutic approaches to treat hypertension.

Natural products, such as traditional Chinese medicines, have been widely used as supplementary therapy for hypertension^5^. Gastrodin is an essential bioactive compound derived from the Chinese herbal medicine *Gastrodia elata* or Tianma. It is widely used in combination with conventional therapy for hypertension and has a significant effect on attenuating increased blood pressure and improving related symptoms, such as dizziness and headache^6^. Moreover, gastrodin is beneficial to elderly patients with refractory hypertension^7^. A series of modern pharmacological studies demonstrated that gastrodin treatment attenuated blood pressure elevation by targeting the renin–angiotensin–aldosterone system and peroxisome proliferator-activated receptor γ in a spontaneous hypertension rat (SHR) model and exhibited a protective effect on retinal ganglion cells in an animal model with acute glaucoma by suppressing microglial activation and neuroinflammation with mediation of microglia^8,9^. These studies provide a basis for the use of gastrodin as a supplementary therapy for hypertension; however, the underlying mechanisms for the antihypertensive effects of gastrodin remain mostly unknown.

Abnormal vasoconstriction and vasodilation can lead to continuous blood pressure elevation^7^, which results in functional and pathological changes in blood vessels, including decreased vascular function and the abnormal proliferation of vascular smooth muscle cells (VSMCs)^10,11^. Gastrodin promotes vasodilation by activating canonical K_ATP_ channels in VSMCs through the protein kinase A (PKA)-dependent signaling pathway^12^; however, the regulatory effects and mechanisms of gastrodin on vascular function and associated pathological changes require further examination.

In this study, we determined the effects of gastrodin on elevated blood pressure, vascular activity, pathological changes, and proliferation of VSMCs in SHRs or isolated primary VSMCs. Putative antihypertensive target genes and signaling pathways associated with gastrodin treatment were identified using network pharmacology and western blot analysis. Gastrodin treatment attenuated the elevation of blood pressure, pulse wave velocity (PWV), and pathological changes in the abdominal aorta. We demonstrated for the first time that gastrodin treatment reduced the proliferation of VSMCs and suppressed the activation of the PI3K/AKT pathway. These findings provide further evidence of the therapeutic efficiency of gastrodin on antihypertension and its underlying mechanisms, which may lead to the use of gastrodin against hypertension in clinic.

## Results

### Analysis of the common target network interactions of gastrodin

**Figure 1:**
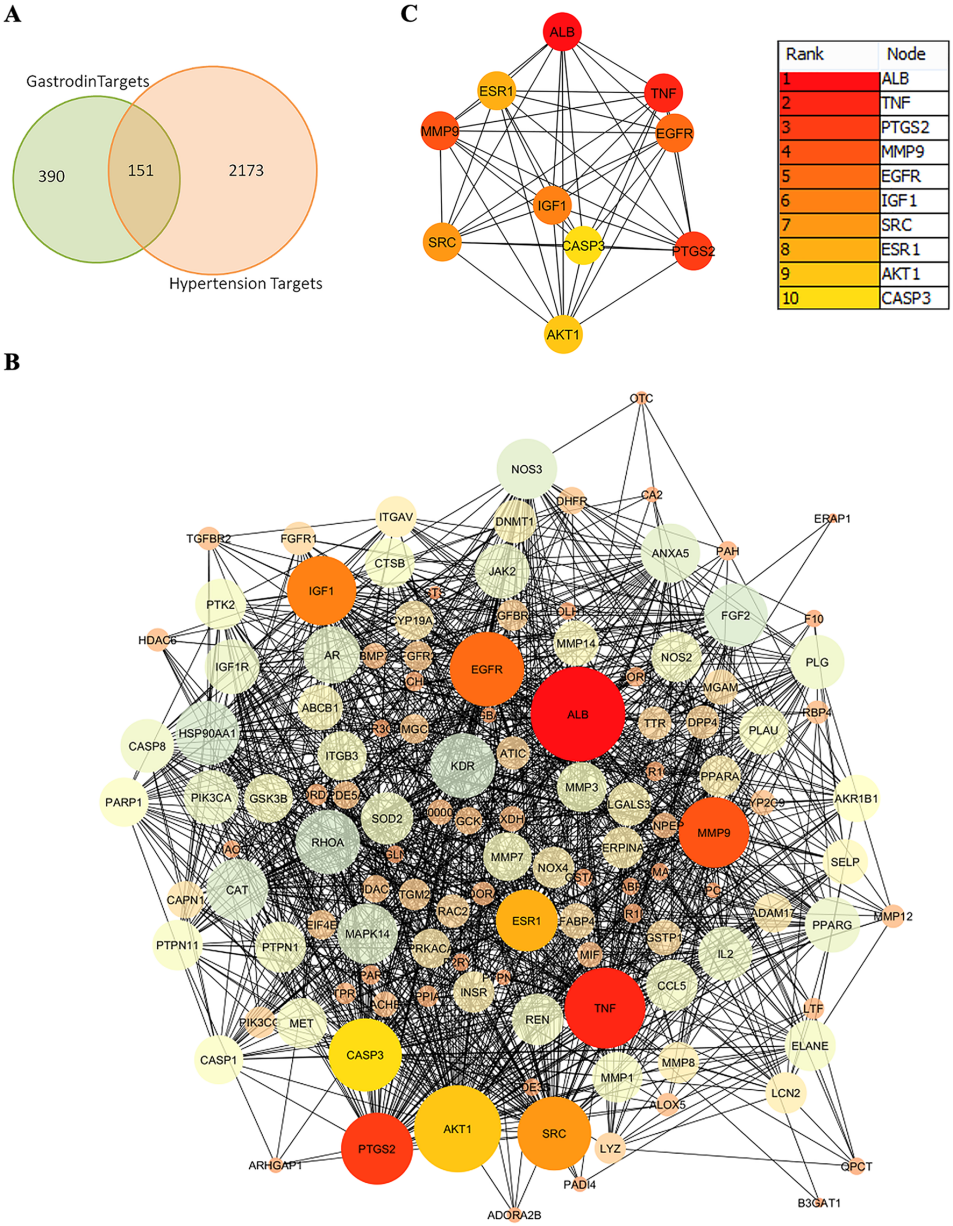
Identification of the common genes and construction of the protein–protein interaction (PPI) network. (**A**) Gastrodin targets and disease-related genes were identified using an intersection of the gastrodin target and hypertension-related genes. (**B**) The PPI network of common genes was constructed using the STRING database. (**C**) The top 10 hub genes are listed. The lines represent PPI, a deeper color of nodes represents higher score values, and the rectangle boxes show the rank and nodes of the hub genes.

The underlying mechanism of gastrodin as an antihypertensive agent was examined using several network pharmacological strategies. A total of 2324 target genes for hypertension were acquired from the DesiGNET, Online Mendelian Inheritance in Man (OMIM), and GeneCards databases, and 541 gastrodin targets were retrieved from PharmMapper. After removing duplicates, Jenn (http://bioinfo.genotoul.fr/jvenn)^13^ was used to identify 151 genes that were considered as potential targets (Fig. 1A). The Search Tool for Recurring Instances of Neighboring Genes (STRING) database was used to construct a PPI network of 151 common target genes, which consisted of 151 nodes and 1419 edges (Fig. 1B). Next, the PPI data were exported into Cytoscape and a protein interaction network (PIN) network was generated. From the protein interaction network and common targets network interaction (PIN/CTNI), the top 10 hub genes from the PPI network were identified using CytoHubba in Cytoscape. As the score decreased, the color of the 10 genes (e.g., TNF, CASP3, ALB, MMP9, EGFR, PTGS2, IGF1, SRC, ESR1, and AKT1) changed from red to yellow (Fig. 1C). These targets were considered important genes that are associated with the efficacy of gastrodin on hypertension.

### Molecular complex detection (MCODE) analysis of PIN/CTNI

**Figure 2:**
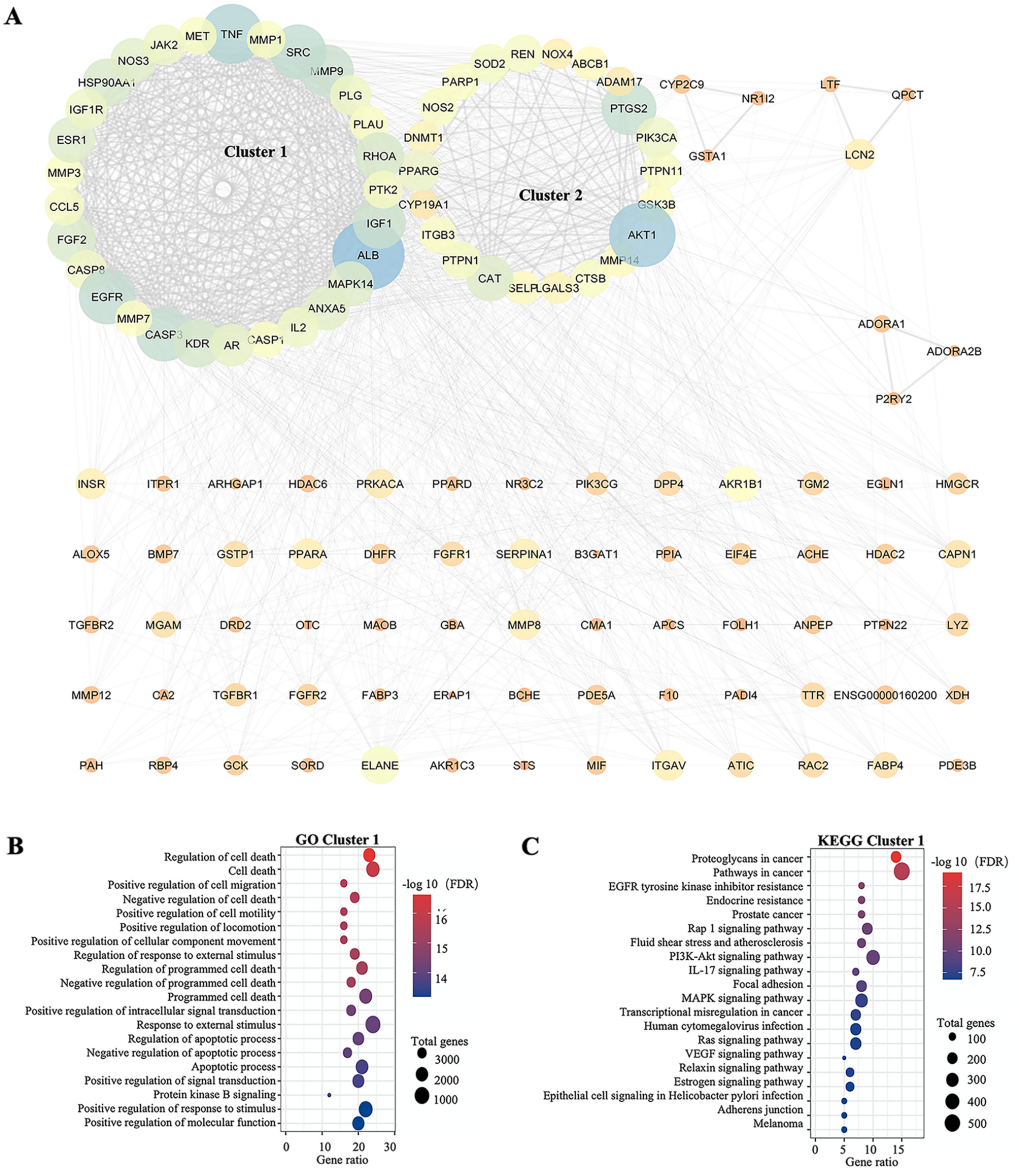

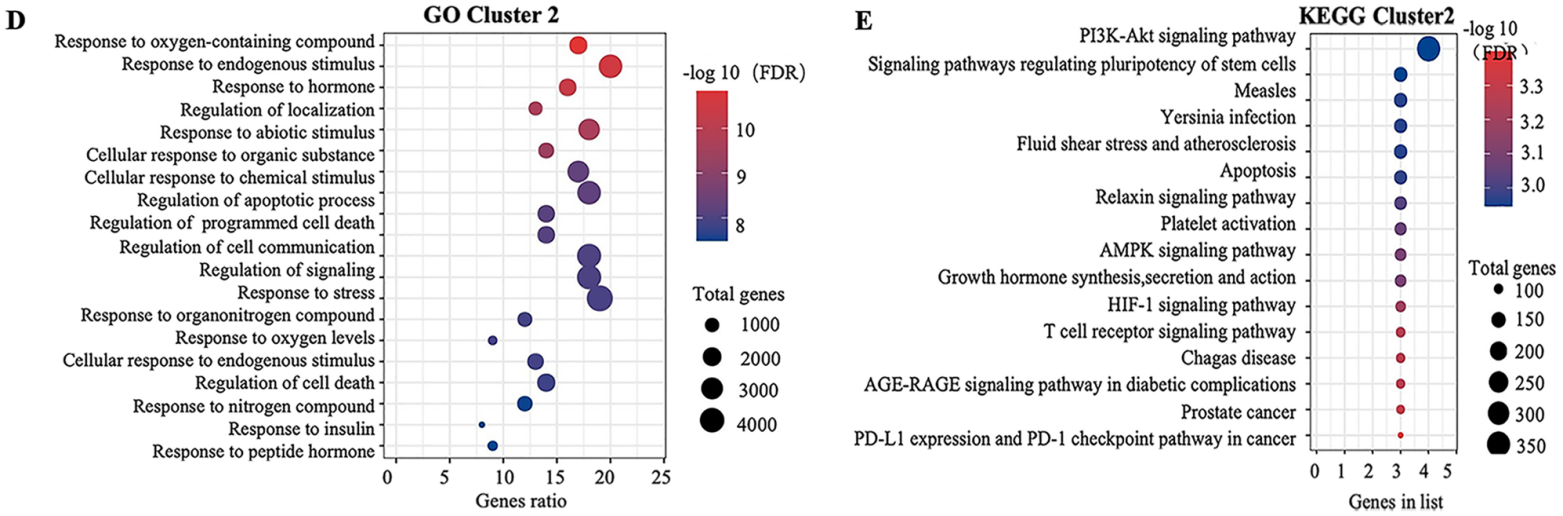
Molecular complex detection analysis of the PPI network of common genes. (**A**) Clusters of common targets network interaction for all common genes are shown. (**B**, **C**) The analyses of the gene ontology (GO) (**B**) and Kyoto Encyclopedia of Genes and Genomes (KEGG) pathways (www.kegg.jp/kegg/kegg1.html) (**C**) of the common genes in cluster 1 are presented. (**D**–**E**) The analyses of GO the processes (**D**) and KEGG (www.kegg.jp/kegg/kegg1.html) pathways (**E**) of cluster 2 are presented.

After examining the functional modules of PIN/CTNI by MCODE, six clusters were identified with a score of more than 3 and two clusters with a score of > 5.6 were selected for gene ontology (GO) and KEGG pathway analyses. The score of cluster 1 was 15.731, and the PIN/CTNI contained 30 nodes and 291 edges (Fig. 2A, cluster 1). In the GO processes, these targets were mainly enriched with cell death regulation, cell migration, apoptotic process, and response to external stimuli (Fig. 2B). KEGG pathway analysis showed that these targets were enriched significantly as cancer pathways and as Rap1, MAPK, and PI3K/AKT signaling pathways (Fig. 2C). The score of cluster 2 was 10.763, and the PIN/CTNI contained 22 nodes and 189 edges (Fig. 2A, cluster 2). Cluster 2 genes were found in multiple GO processes, such as regulation of apoptosis, regulation of stress, regulation of programmed cell death, and response to endogenous stimuli (Fig. 2D). The KEGG analysis was enriched with apoptosis, measles, and the AMPK and PI3K/AKT signaling pathways (Fig. 2E).

### Identification of enriched biological functions and pathways

**Figure 3:**
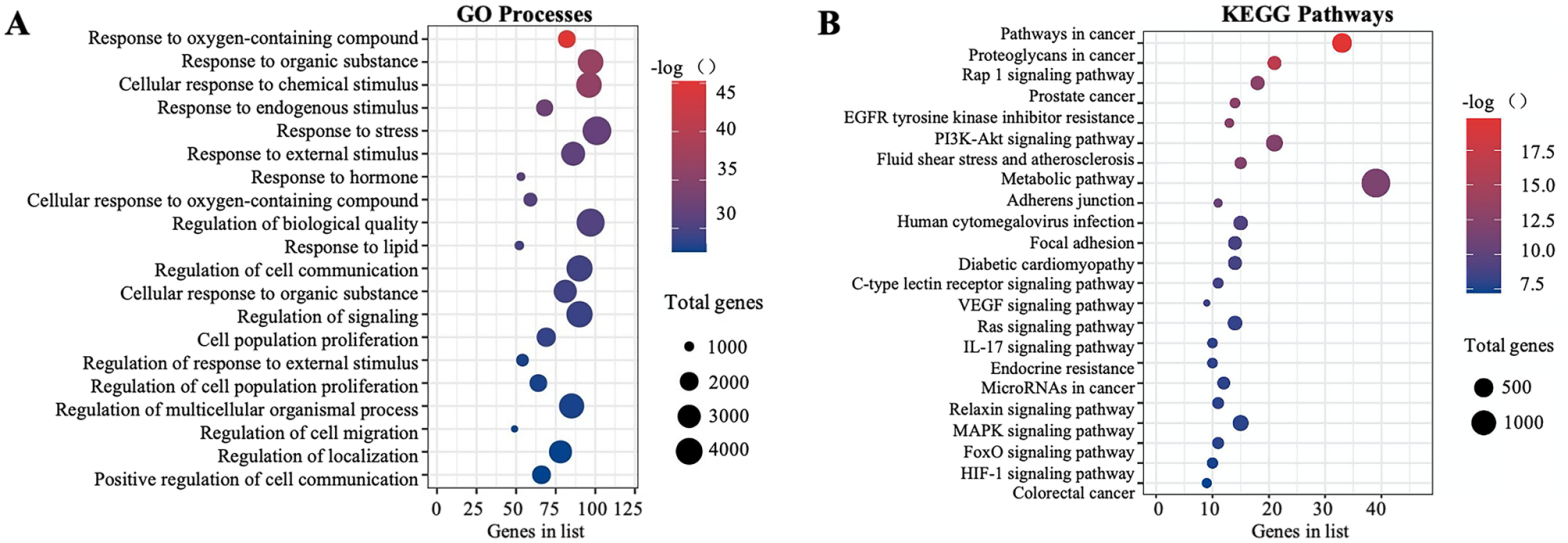
Enrichment analyses by Gene Ontology (GO) and Kyoto Encyclopedia of Genes and Genomes (KEGG). (**A**) GO enrichment analysis of all common genes. The x-axis represents the gene number, the y-axis represents the term, and the color indicates –log10 (FDR). (**B**) KEGG pathway analysis of all common genes. The node size represents the gene number and the color indicates –log10 (FDR) (www.kegg.jp/kegg/kegg1.html).

In the GO analysis, 567 GO-enriched biological processes were identified; the top 20 GO processes that were visualized in a bubble diagram (Fig. 3A) included response to oxygenated compounds, response to stress, response to external stimuli, cell communication regulation, cell proliferation population, cell migration regulation, regulation of localization, response to hormone, and regulation of signaling. In summary, we speculated that the mechanisms of gastrodin as an antihypertensive medicine simultaneously involved these biological processes and molecular functions.

Using KEGG analysis, 157 enriched pathways were identified and the top 20 pathways according to FDR value are shown in Fig. 3B. These included pathways associated with cancer, focal adhesion, diabetic cardiomyopathy, and the PI3K/AKT, VEGF, Ras, and MAPK signaling pathways. The results indicate that these signaling-related pathways are associated with the effects of gastrodin against hypertension.

### Gastrodin attenuates blood pressure elevation in SHRs

**Figure 4:**
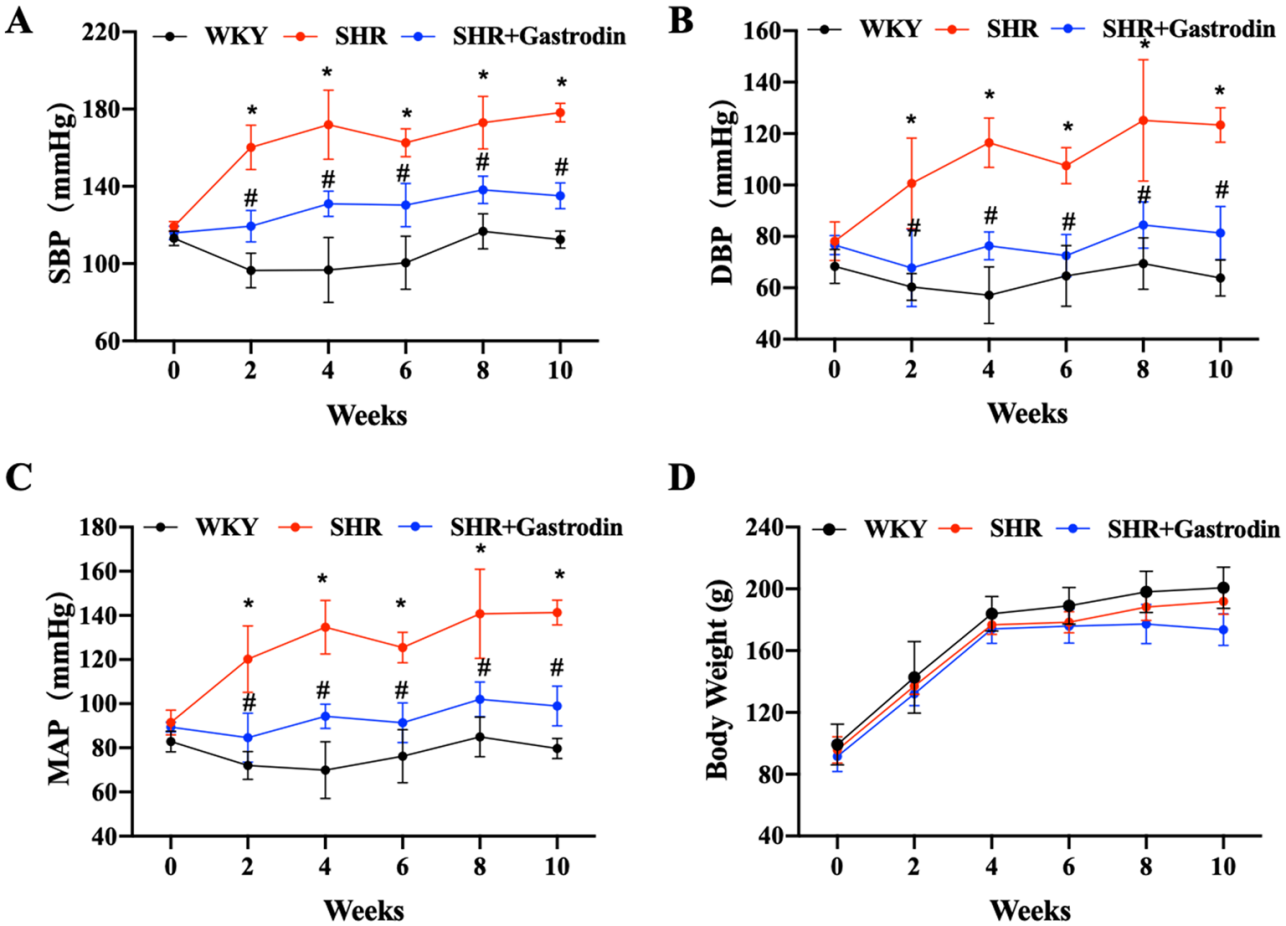
Gastrodin attenuates elevation of blood pressure in spontaneous hypertensive rats (SHRs). Blood pressure, including (**A**) systolic blood pressure (SBP), (**B**) diastolic blood pressure (DBP), and (**C**) mean arterial pressure (MAP) were monitored using a tail cuff plethysmograph and the CODA noninvasive blood pressure system. (**D**) The body weight of the rats from each group was recorded. Data are presented as the mean ± SD; n = 5 for each group. *p < 0.05 versus The WKY group, #*p* < 0.05 versus the SHR group.

Blood pressure measurements indicated that systolic blood pressure (SBP; Fig. 4A), diastolic blood pressure (DBP; Fig. 4B), and mean arterial pressure (MAP; Fig. 4C) in SHRs were significantly increased (Fig. 4A–C; *P < 0.05, vs. WKY group), which were all attenuated following gastrodin treatment (Fig. 4A–C; #*P* < 0.05, vs. SHR group). In addition, gastrodin treatment did not cause a body weight change in the SHR model (Fig. 4D; all *P* > 0.05).

### Gastrodin alleviates vascular function and pathological changes in SHRs

**Figure 5:**
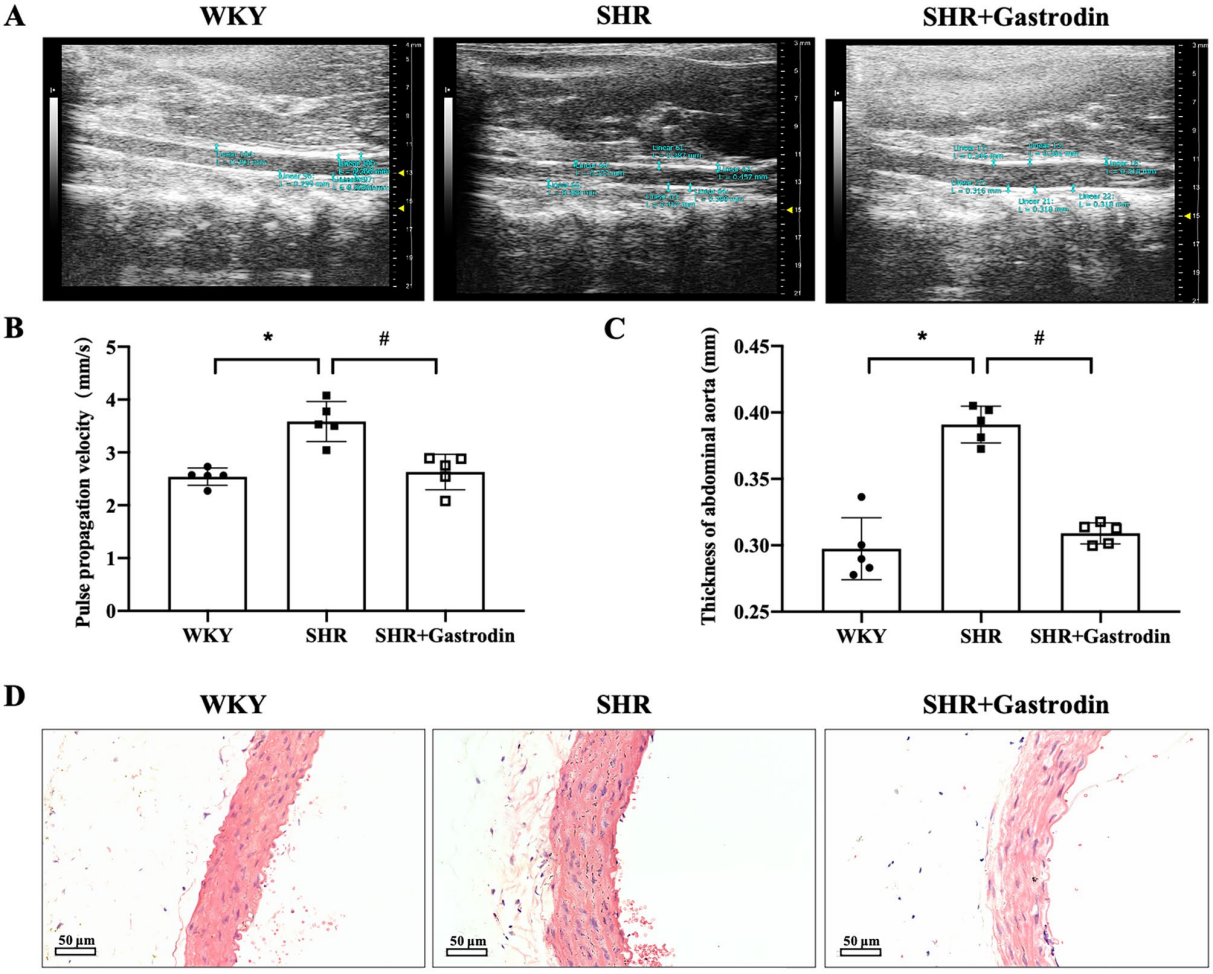
Gastrodin alleviates changes in the vascular function and pathology of the abdominal aorta in SHRs. The effects of gastrodin treatment on the vascular function and pathology of the abdominal aorta in SHRs were determined by animal ultrasound and hematoxylin and eosin (H&E) staining (**A**–**C**). Representative ultrasound images of each group are shown (**A**). The pulse wave velocity (**B**) and thickness (**C**) of the abdominal aorta of the rats from each group were analyzed by ultrasound. (**D**) Representative H&E staining images of the abdominal aorta of the rats from each group were captured using a microscope at a magnification of 400 × . Data are presented as mean ± SD; n = 5 for each group. *p < 0.05 versus The WKY group, #*p* < 0.05 versus the SHR group.

An analysis of the abdominal aorta of rats from each group revealed a significantly enhanced PWV in the SHRs (Fig. 5A,B; *P < 0.05, vs. WKY group), which was attenuated following gastrodin treatment (Fig. 5A,B; #*P* < 0.05, vs. SHR group). Moreover, the abdominal aorta thickness measured by ultrasound analysis (Fig. 5C) was increased and pathological changes were observed following Hematoxylin and eosin (H&E) staining (Fig. 5D) in the aorta of rats from each group (*P < 0.05, vs. The WKY group), which were alleviated following gastrodin treatment (#*P* < 0.05, vs. SHR group).

### Gastrodin inhibits the proliferation of Ang II-stimulated VSMCs

**Figure 6:**
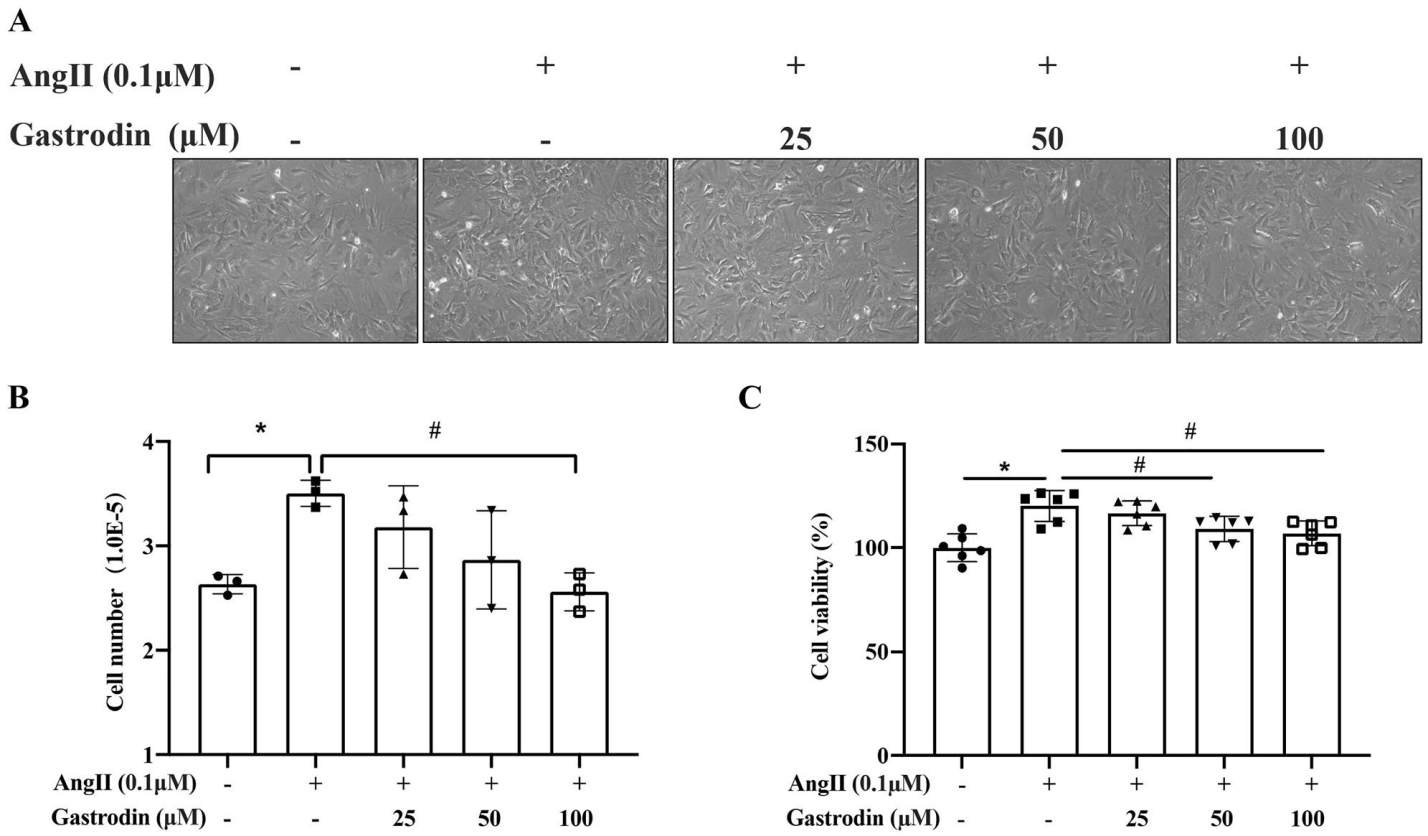
Gastrodin attenuates the proliferation of Angiotensin II (Ang II) -stimulated vascular smooth muscle cells (VSMCs). The effects of gastrodin treatment on cell proliferation. (**A**) Phase contrast light microscopy showing the growth of Ang II-stimulated VSMCs after gastrodin treatment (200 ×). (**B**) The cell number of Ang II-stimulated VSMCs after gastrodin treatment was determined by trypan blue staining. (**C**) The cell viability of Ang II-stimulated VSMCs after gastrodin treatment for 24 h was determined by cell counting kit-8 analysis. Data were normalized to the viability of untreated control cells designated 100%. Data are presented as the mean ± SD. *p < 0.05 versus control group, #*p* < 0.05 versus Ang II group.

**Figure 7:**
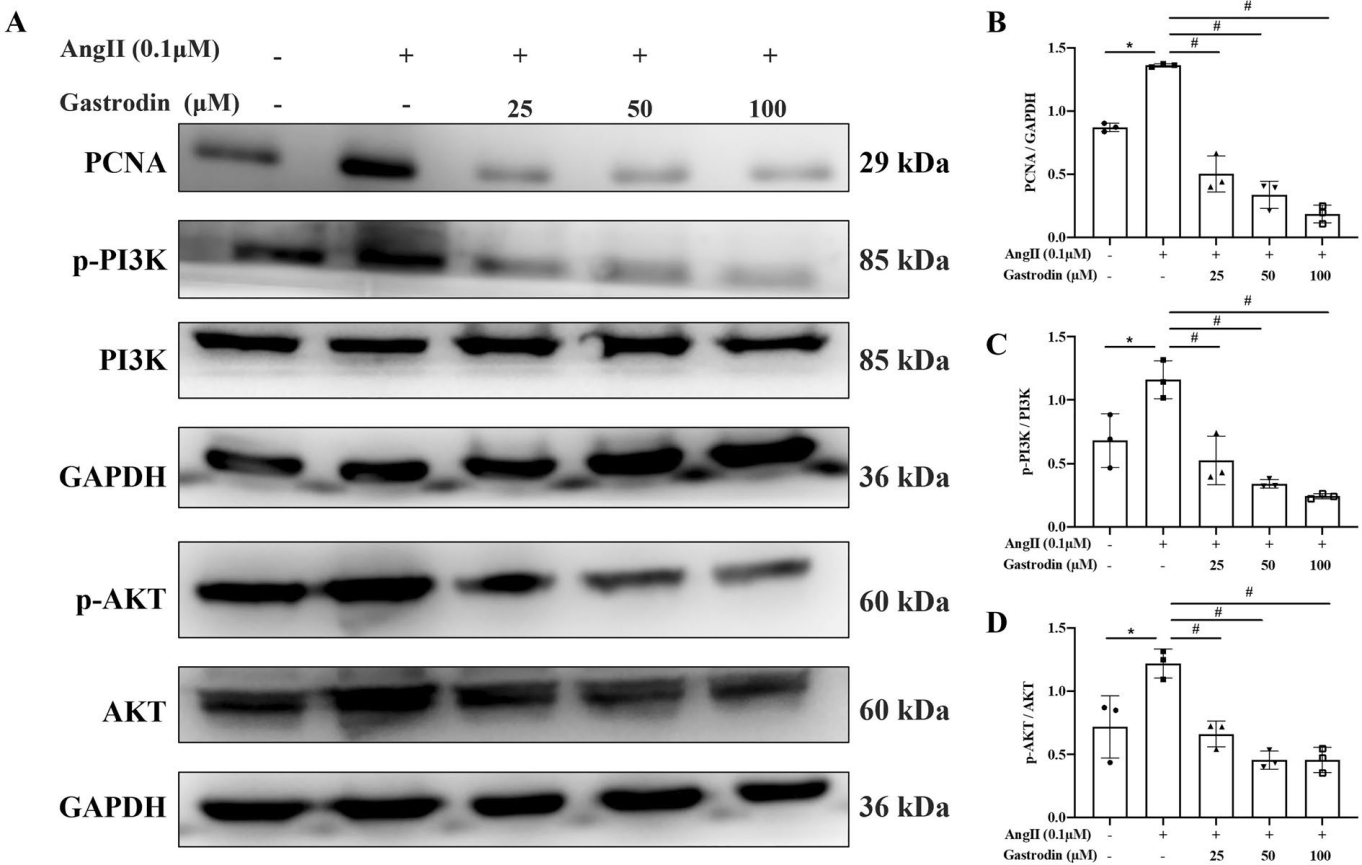
Gastrodin suppresses the activation of the PI3K/AKT pathway. The effects of gastrodin treatment on the PI3K/AKT pathway. (**A**–**D**) The expression of PCNA, p-PI3K and p-AKT protein in Ang II-stimulated VSMCs after gastrodin treatment were determined by western blot analysis and (**A**) the representative images are shown in the left panel. (**B**–**D**) The intensity of the bands was quantitated using ImageJ software and the levels of PCNA was normalized to GAPDH, p-PI3K and p-AKT were normalized to PI3K and AKT respectively, and GAPDH was used as a loading control. The original blots were presented in Supplementary Figure S2, S3 and S4. Data are presented as the mean ± SD. *p < 0.05 versus control group, #*p* < 0.05 versus Ang II group.

To determine the inhibitory effect of gastrodin on the proliferation of Ang II-stimulated VSMCs, primary VSMCs were isolated and confirmed by immunofluorescence staining with α-SMA antibody (Supplementary Figure S1A). A CCK-8 analysis revealed that the viability of VSMCs was not affected by 0–400 μM gastrodin (Supplementary Fig. S1B), but was significantly stimulated by 0.1 μM and 1 μM Ang II (Supplementary Fig. S1C). Therefore, lower concentrations of Ang II (0.1 μM) and gastrodin (25, 50, or 100 μM) were selected for subsequent studies. Interestingly, cell confluence (Fig. 6A), cell number (Fig. 6B), and cell viability (Fig. 6C) were all significantly increased in Ang II-stimulated VSMCs (*P < 0.05, vs. untreated cells), whereas gastrodin treatment significantly attenuated the enhanced cell proliferation of Ang II-stimulated VSMCs (#*P* < 0.05, vs. Ang II). Consistently, the expression of the cell proliferation biomarker PCNA was significantly up-regulated in Ang II-stimulated VSMCs, whereas it was downregulated following gastrodin treatment of Ang II-stimulated VSMCs (Fig. 7A,B; *P < 0.05, vs. untreated cells; #*P* < 0.05, vs. Ang II). Taken together, gastrodin inhibited the proliferation of Ang II-stimulated VSMCs.

### Gastrodin suppresses activation of the PI3K/AKT pathway

Based on the above functional and mechanistic studies, western blot analysis was done to evaluate the regulatory effect of gastrodin treatment on PI3K/AKT pathway activation. As shown in Fig. 7A and Fig. 7C,D, the levels of p-PI3K/PI3K and p-AKT/AKT were significantly increased in Ang II-induced VSMCs, but were attenuated following gastrodin treatment (*P < 0.05, against untreated cells; #*P* < 0.05, vs. Ang II). However, the stimulation of Ang II and combination of gastrodin treatment didn’t affect the expression of PI3K and AKT (Fig. 7A and Fig. 7C,D, *P* > 0.05).

## Discussion

Hypertension is a common chronic disorder and a major risk factor for cerebrovascular and cardiac vascular disorders that can lead to stroke, heart attack, and kidney disease^1,2,14^. Despite the increasing awareness of hypertension and the use of antihypertensive drug combinations, current treatment for hypertension remains unsatisfactory. Therefore, it is necessary to develop alternative approaches to hypertension therapy^15,16^. Gastrodin, which is the primary bioactive ingredient in the Chinese herb Tianma, is widely used as an alternative therapy with significant antihypertensive and neuroprotective effects^6–9,17^. In a clinical study, gastrodin significantly reduced the blood pressure of patients with hypertension^6^; however, its regulatory effect and underlying mechanism, particularly on vascular function, needs to be explored further.

Network pharmacology has been used extensively to determine the complex mechanisms of traditional Chinese medicine^18^. In the present study, we identified 390 predicted potential targets of gastrodin and 2173 hypertension-related genes. An intersection analysis between the gastrodin targets and hypertension-related genes identified 151 common potential targets. Ten hub genes were identified from the PIN/CTNI network including TNF, CASP3, ALB, MMP9, EGFR, PTGS2, IGF1, SRC, ESR1, and AKT1. Of these, TNF, MMP9, and AKT1 have been reported to be involved in hypertension^19–21^ and considered important genes that function in the effect of gastrodin on hypertension. However, the regulatory effect of gastrodin on these proteins and the potential binding of gastrodin with these proteins needs to be examined in future studies.

Functional modules of PIN/CTNI identified two clusters. Interestingly, multiple signaling pathways were associated with the genes of cluster 1, cluster 2, and all genes. Interestingly, three different enrichment analyses indicated that the PI3K/AKT signaling pathway was substantially enriched; which prompted us to probe the regulatory function of gastrodin treatment on PI3K/AKT pathway activation.

Next, we examined the vascular function following gastrodin treatment of SHRs. Gastrodin attenuation of SBP, DBP, and MAP provided an experimental basis for the antihypertensive effect of gastrodin; however, further animal studies should be done to explore the therapeutic efficacy of gastrodin on hypertension. Abnormal vasoconstriction and vasodilation can lead to persistent and prolonged blood pressure elevation, which results in vascular dysfunction and pathological changes^11,12^. Gastrodin treatment significantly promoted vasodilation through a PKA-dependent signaling pathway^12^. Our study revealed that gastrodin treatment significantly decreased PWV and abdominal aorta thickness in SHRs, suggesting that gastrodin attenuates vascular dysfunction and pathological changes of the abdominal aorta in SHRs. Future studies are needed to evaluate the effects of gastrodin treatment on damage to major organs.

VSMCs, which represent the major cell type in the arteries, are responsible for vascular tone and exhibit remarkable plasticity. In healthy arteries, most VSMCs can regulate vascular tone and hemodynamic balance by maintaining a contractile phenotype in the media^22^. However, during a physiological or pathological state, VSMCs can switch from a contractile phenotype to a synthetic, proliferative, and migrating status, which results in vascular remodeling and dysfunction^22^. In the present study, the attenuated growth of VSMCs and the downregulated expression of PCNA and Ang II-stimulated VSMCs following gastrodin treatment further confirmed that gastrodin suppressed VSMC growth; however, the regulatory effect of gastrodin treatment on aortic fibrosis, inflammation, and other pathological changes requires further study.

Based on a network pharmacology analysis, gastrodin treatment attenuated the increase of p-PI3K/PI3K and p-AKT/AKT levels in Ang II-stimulated VSMCs. This suggests that a potential underlying mechanism of gastrodin treatment is the suppression of Ang II-stimulated VSMCs growth. However, several enriched signaling pathways, including ERK, AMPK, and FoxO^23–25^, have been reported to play vital functional activities in hypertensive vascular disease; however, this requires further verification. Moreover, except for the above enriched pathways, multiple cancer-associated pathways (e.g., cancer, proteoglycans in cancer, prostate cancer) were significantly enriched, suggesting/the potential antitumor effects of gastrodin, which is consistent with previous results as an adjuvant treatment for various cancers^26–29^. However, the details of the anticancer effects of gastrodin and its underlying mechanism of action need to be examined.

**Figure 8:**
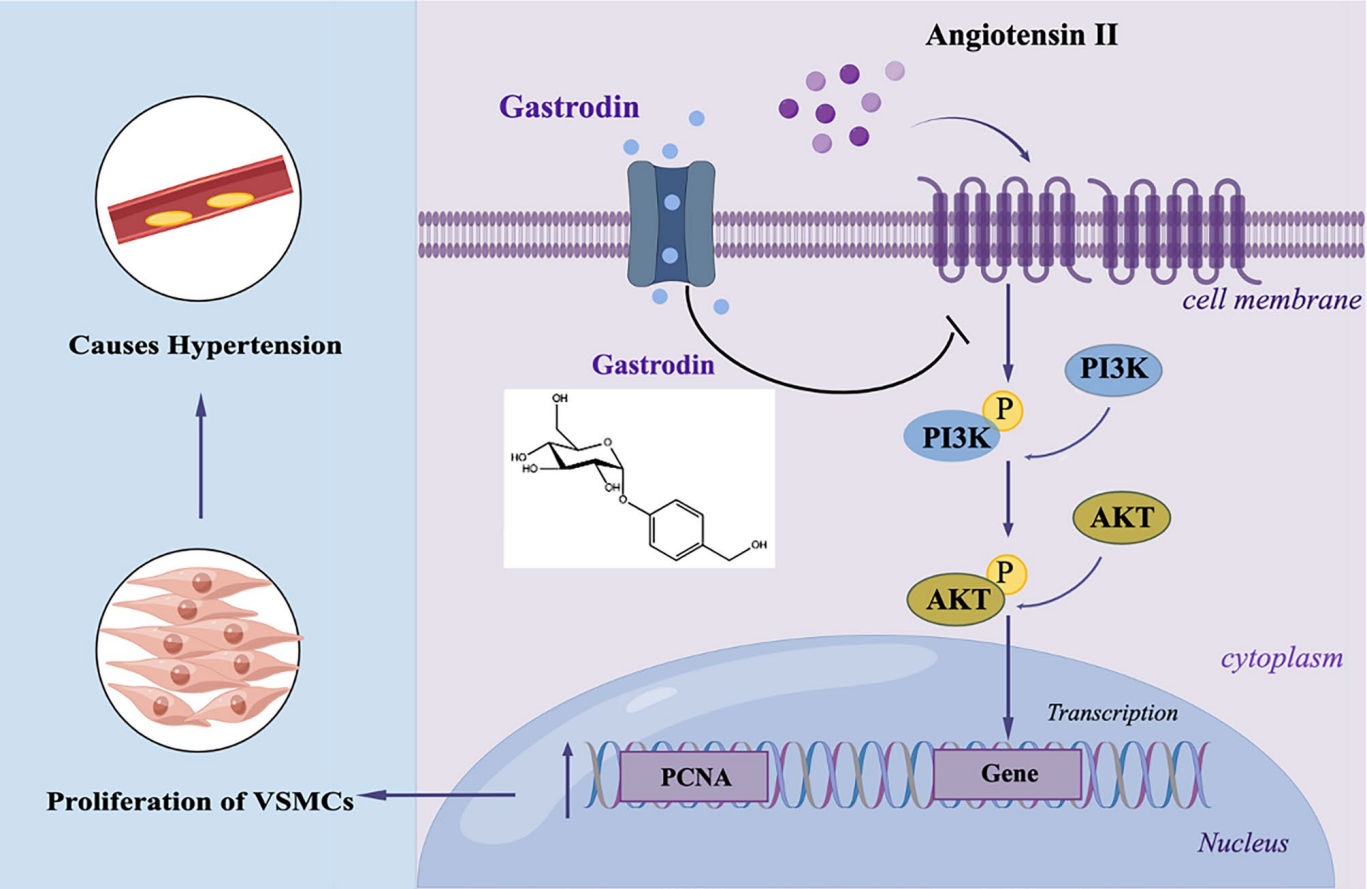
Graphical presentation of the antiproliferative effect of gastrodin by suppressing PI3K/AKT pathway activation in VSMCs. Ang II mediates the phosphorylation of PI3K and phosphorylated PI3K activates AKT through phosphorylation. AKT promotes cell survival by inhibiting apoptosis through phosphorylation, which leads to the proliferation of VSMCs during the development of hypertension. Gastrodin is an essential bioactive compound derived from the Chinese herbal medicine *Gastrodia elata* or Tianma. Gastrodin treatment alleviates vascular dysfunction and pathological changes of the abdominal aorta in SHRs. Moreover, gastrodin suppresses the growth of Ang II-stimulated VSMCs by attenuating multiple signaling pathways, including the PI3K/AKT pathway. These may be the essential underlying mechanism that warrants the clinical use of gastrodin for hypertension. Prepared by Figdraw.

In summary, the mechanisms of gastrodin antihypertension are presented in Fig. 8 and we demonstrated that gastrodin treatment attenuates blood pressure elevation, vascular dysfunction, and the pathological changes observed in the abdominal aorta of SHRs and suppresses the growth of Ang II-stimulated VSMCs through mediation of multiple signaling pathways, including the PI3K/AKT pathway. These may be the essential underlying mechanisms that support the clinical use of gastrodin for hypertension.

## Materials and methods

### Reagents and antibodies

The antibodies p-PI3K (cat no.4228) PI3K (cat no. 4257), p-AKT (cat no. 4060), AKT (cat no. 4691), and α-SMA (cat no.19245) were obtained from Cell Signaling Technology (Beverly, MA, USA). Ang II (cat no. Ab120183) were acquired from Abcam (Cambridge, MA, USA). The antibody against glyceraldehyde 3 phosphate dehydrogenase (GAPDH; cat no. L1021) and the CCK-8 assay kit (cat no. ATVK21031) were purchased from Abbkine (Wuhan, Hubei, China). Anti-PCNA (cat no.48728) and goat anti-rabbit IgG secondary antibodies (cat no. L3012) were obtained from Signalway Antibody (College Park, MD, USA). Dulbecco’s Modified Eagle Medium (DMEM; cat no. C11995500BT), fetal bovine serum (FBS; cat no. 10099141C), 0.25% trypsin EDTA (cat no. 25200072), and the Pierce Bicinchoninic acid (BCA; cat no. UL298159) protein assay kit were purchased from Thermo Fisher Scientific (Waltham, MA, USA). Eosin Y staining solution (cat no. G1100) and Cole’s hematoxylin solution (cat no. G1140) were obtained from Solaribo (Beijing, China).

### Animals

Female SHRs (n = 10; 4-week-old; weight, 93 ± 9 g) and Wistar Kyoto (WKY) rats (n = 5; 4-week-old; weight, 99 ± 13 g) were acquired from Beijing Vital River Laboratory Animal Technology Co., Ltd. (Beijing, China) and were grown in the Animal Center of Fujian University of Traditional Chinese Medicine. Animal welfare and experimental protocols were performed in accordance with the ARRIVE guidelines and were applied in strict accordance with the guidelines of the Guide for the Care and Use of Laboratory Animals of the USA. The animal experimental protocol for this study was approved by the Animal Care and Use Committee of Fujian University of Traditional Chinese Medicine (approval no. 2021132).

All rats were housed in rooms with a 12-h light/dark cycle at 22 °C–26 °C and a relative humidity of 50%–60%. They had free access to drinking water and standard laboratory chow. The rats were acclimated to laboratory conditions for 2–3 days. Thereafter, the SHRs were grouped into SHR and SHR + gastrodin groups (each group, n = 5), whereas the WKY rats (n = 5) were considered WKY control animals.

### Preparation of gastrodin

Gastrodin (cat no. B21243) was obtained from Shanghai Yuanye Bio-Technology Co., Ltd. (Shanghai, China). For the animal experiments, gastrodin powder was dissolved in double-distilled water (ddH_2_O) to obtain a concentration of 3.5 mg/kg/day, based on the average body weight of the rats^8^. For cell experiments, a 1 M stock solution was prepared by dissolving gastrodin in ddH_2_O before completely dissolving it with medium before use.

### Blood pressure measurement

Before the experiments, blood pressure was measured by a tail cuff plethysmograph using the CODA™ noninvasive blood pressure system (Kent Scientific; Torrington, CT, USA) according to the manufacturer’s guidelines and as described previously^30^, once every two weeks following gastrodin treatment.

### Ultrasound measurement

The abdominal aortic thickness and PWV were determined using a Vevo 2100 Ultrasound Machine (VisualSonics; Toronto, Ontario, Canada) according to the manufacturer’s instructions and as previously described^31^.

### H&E staining

After 10 weeks of gastrodin therapy, abdominal aortic tissue was collected from each rat and fixed in 4% paraformaldehyde (cat no. BL539A; Biosharp; Hefei, Anhui, China), embedded in paraffin, sectioned at 4-µm thickness, and stained with H&E after rehydration with gradient ethanol. The slides were visualized under a Leica DM4000B intelligent automated optical microscope (Leica; Weztlar, Germany) at 400 × magnification.

### Isolation of primary vascular smooth muscle cells

The VSMCs were isolated as previously described^32^. After isoflurane anesthesia, the abdominal aorta was swiftly excised and washed with saline buffer containing 1.5 mM CaCl_2_-HEPES. Thereafter, the aorta was cut longitudinally and the endothelial cells were carefully stripped with a cotton swab in Ca^2+^-free HEPES-buffered salt solution. An enzyme mixture containing collagenase I (cat no. 2256525; 1,750 units), papain (cat no. P4762; 9.5 units), and BSA (cat no. V900933; 2.0 mg) was used to digest the abdominal aorta for 30 min at 37 °C in an incubator containing 5% CO_2_. The isolated primary VSMCs were seeded into DMEM containing 10% FBS, 100 U/mL of 1% penicillin, and 100 g/mL of streptomycin (cat no. SV30010; Hyclone; Logan, UT, USA).

### Immunofluorescence analysis

Isolated primary VSMCs were cultured in glass-bottom plates at a density of 8000 cells/well. After 24 h, the cells were fixed for 15 min with 4% paraformaldehyde, washed three times with PBS (cat no. 2246306; VivaCell Biotechnology; Denzlingen, Germany), permeabilized for 10 min with 0.2% Triton X-100 (cat no. T8200; Solaribo), and placed for 1 h in a blocking buffer containing 5% BSA, 10% goat serum, and 85% PBS. The cultured cells were incubated with antibodies against α-SMA (1:200) at 4 °C overnight, washed thrice using PBS, and incubated with secondary anti-rabbit antibody (1:400) for one hour in the dark at room temperature. The cells were washed three times with PBS, incubated with Hoechst (cat no. 33342; Solaribo) for 10 min, and observed using a confocal microscope (PerkinElmer Inc.; Waltham, MA, USA).

### Analysis of cell confluence and counting of cell number

VSMCs were treated with Ang II and various concentrations of gastrodin for 24 h. Microscopy (Leica) at 200 × magnification was used to observe cell confluence. The cells were digested and cell number was determined using a Countstar Automated Cell Counter (Shanghai, China) after staining with 0.4% Trypan Blue (cat no. C0040; Solaribo).

### CCK-8 assay

Primary isolated VSMCs were cultured in 96-well plates for 24 h, followed by treatment with Ang II and/or various concentrations of gastrodin for 24 h. Then, 10 μL of CCK-8 solution (cat.no. ATVK21031; Abbkine) was added to the wells and incubated at 37 °C for another 2 h. The absorbance at 450 nm was measured using a microplate reader (Thermo Fisher Scientific) based on the manufacturer’s guidelines. The viability of the untreated cells was designated as 100%.

### Screening of gastrodin and hypertension targets

The gastrodin structure in SDF format was uploaded into the PharmMapper Server^33,34^ and SwissTarget Prediction^35,36^ (http://www.swisstargetprediction.ch/) to obtain the targets. The scoring criteria differed between the databases. The target genes of gastrodin were selected based on a fit value of > 0.5 in the PharmMapper Server and a probability value > 0 for the SwissTarget Prediction. The disease-associated genes were collected from the DesiGNET, GeneCards^37^, and OMIM databases^38^ using hypertension and high blood pressure as keywords. The gene names were then unified by UniProt and duplicate genes were removed.

### Protein–protein interaction data

The common target genes for gastrodin and hypertension were obtained and mapped using Venn diagrams. The protein–protein interaction (PPI) data were extracted using the STRING database^39^ and selected with a confidence score > 0.4 for the construction of the PIN/CTNI.

### Network construction and analysis

The PPI data exported from the STRING database were uploaded into Cytoscape 3.6.0 and visualized and analyzed after hiding the disconnected nodes. The Network Analyzer tool was used to evaluate the topological attributes of the degree values. We used CytoHubba^40^ in Cytoscape to analyze the PPI network by maximal clique centrality and identified the hub genes. Thereafter, the MCODE algorithm^41^ was used to cluster the PIN/CTNI network.

### Analysis of GO functions and KEGG pathways

The DAVID database (version 6.8) was used to analyze the GO and KEGG pathways^42–45^(www.kegg.jp/kegg/kegg1.html). Outcomes were visualized using the R dot chart package.

### Western blot analysis

Cells were placed in a lysis solution (Beyotime Biotech, Inc., Shanghai, China) along with a protease inhibitor before centrifugation at 12,000 g at 4 °C for 20 min. BCA was used to calculate total protein concentrations. Equivalent amounts of protein were separated by SDS-PAGE and transferred to PVDF membranes (cat no. 0000199759) (Merck Millipore; Darmstadt, Germany). The membranes were placed into blocking buffer for 2 h at room temperature and incubated with primary antibodies (p-AKT, AKT, p-PI3K, PI3K, PCNA; 1:1000 dilution) or GAPDH antibody (1:5000 dilution) overnight at 4 °C. In addition, the membranes were washed three times with TBST (cat no. T1081; Solaribo) before incubation with secondary antibody at a dilution of 1:5000 for 1 h at 37 °C. The blots were developed using a chemiluminescence kit (cat no. P0018M, Beyotime Biotech, Inc.) and visualized using the Bio-Rad Chemi Doc XRS imaging system (Bio-Rad; Hercules, CA, USA). ImageJ software was used to quantitate band intensities. The expression of GAPDH was used as an internal control.

### Statistical analysis

The experimental data were presented as the mean ± standard deviation and analyzed by SPSS 26.0 (IBM Corp., Chicago, IL, USA). As for the data met normal distribution One-way analysis of variance was used to compare the differences among three or more groups followed by Bonferroni analysis when the variance was chi-square, while followed by Games Howell analysis when the variance was not chi-square. As for the data didn’t meet normal distribution, non-parametric analysis followed by Kruskal–Wallis test was used to comparing the difference among three or more groups. Statistical significance was assigned to differences with *p* values < 0.05. GraphPad Prism (version 8; GraphPad Software; San Diego, CA, USA) was employed to illustrate the graphs.

## Supplementary Information

Supplementary Figure S1.

Supplementary Figure S2.

Supplementary Figure S3.

Supplementary Figure S4.

## Data Availability

The datasets used and/or analyzed during the current study are available from the corresponding author on reasonable request.

## Abbreviations

α-SMA: Alpha-smooth muscle actin
Ang II: Angiotensin II
BCA: Bicinchoninic acid
BSA: Bovine serum albumin
CCK-8: Cell counting Kit-8
DBP: Diastolic blood pressure
DAVID: Database for annotation visualization and integrated discovery
DEPs: Different expression proteins
ddH_**2**_**O**: Double distilled water
DMEM: Dulbecco’s modified eagle medium
ECM: Extracellular matrix
FBS: Fetal bovine serum
GO: Gene Ontology
HBSS: HEPES-buffered salt solution
GAPDH: Glyceraldehyde 3‑phosphate dehydrogenase
H&E: Hematoxylin and Eosin
IF: Immunofluorescence
KEGG: Kyoto encyclopedia of genes and genomes
MCC: Maximal clique centrality
MAP: Mean arterial pressure
MCODE: Molecular complex detection
OMIM: Online mendelian inheritance in man
PPARγ: Peroxisome proliferator-activated receptor γ
PKA: Protein kinase A
PBS: Phosphate-buffered saline
PVDF: Polyvinylidene fluoride membrane
PIN: Protein-interaction network
PWV: Pulse wave velocity
PPI: Protein–Protein interaction
PIN/CTNI: Protein interaction network and common targets network interaction
RAAS: Renin angiotensin-aldosterone system
SHRs: Spontaneously hypertensive rats
STRING: Search tool for recurring instances of neighboring genes
SBP: Systolic blood pressure
TSV: Tab-separated value
VSMCs: Vascular smooth muscle cells
WKY: Wistar Kyoto
