# Supplementary material for: Based on network pharmacology, gastrodin attenuates hypertension-induced vascular smooth muscle cell proliferation and PI3K/AKT pathway activation
Source: Sci Rep. 2023 Jul 26;13:12140. doi: 10.1038/s41598-023-39202-6 (PMC10372005; doi:10.1038/s41598-023-39202-6)
Supplement: Supplementary file 1 — Supplementary Figure S1. [file 41598_2023_39202_MOESM1_ESM.pdf]

Supplementary figure

Figure S1. Identification of the VSMCs and the cell viability of GAS or AngII treatment.

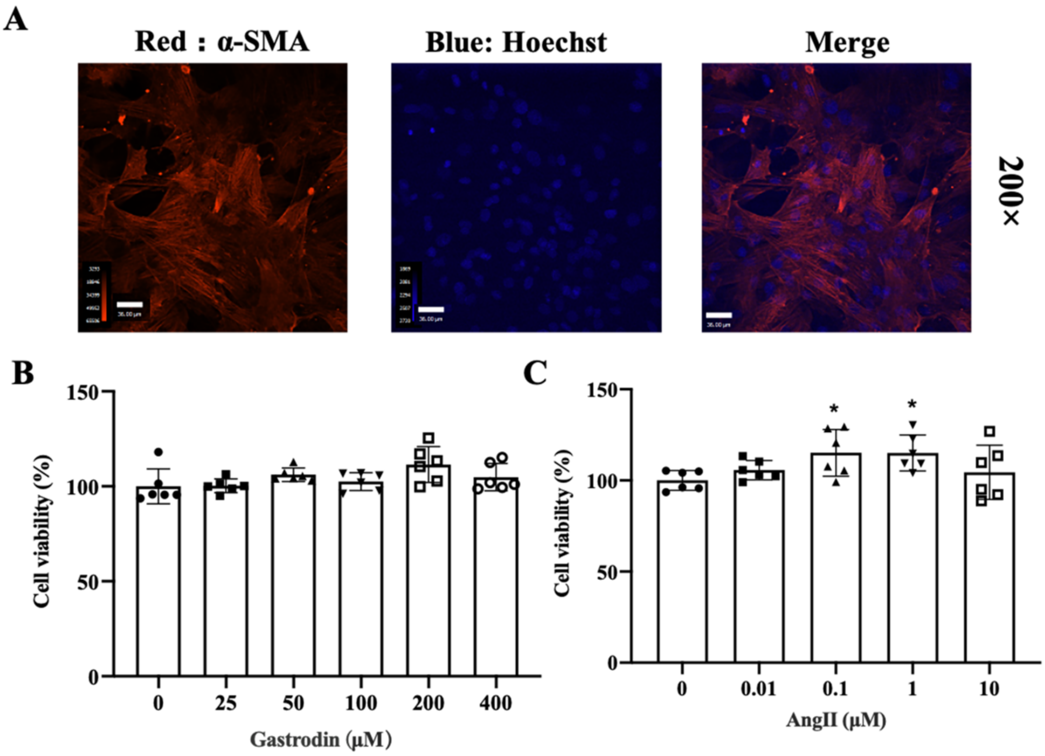

We next investigate whether Gastrodin treatment inhibits VSMCs proliferation. (A) The primary VSMCs were isolated and identified by immunofluorescence staining against α-SMA antibody using confocal microscopy at a magnification of 200×. (B, C) The cell viability of VSMCs after (B) Gastrodin (0, 25, 50, 100, 200, 400 μM) or (C) Ang II (0, 0.01, 0.1, 1, 10 μM) treatment for 24 h was determined by CCK-8 analysis. Data were normalized to the viability of untreated control cells and set as 100%.
