# Supplementary figures and images for: Based on network pharmacology, gastrodin attenuates hypertension-induced vascular smooth muscle cell proliferation and PI3K/AKT pathway activation
Source: Sci Rep. 2023 Jul 26;13:12140. doi: 10.1038/s41598-023-39202-6 (PMC10372005; doi:10.1038/s41598-023-39202-6)

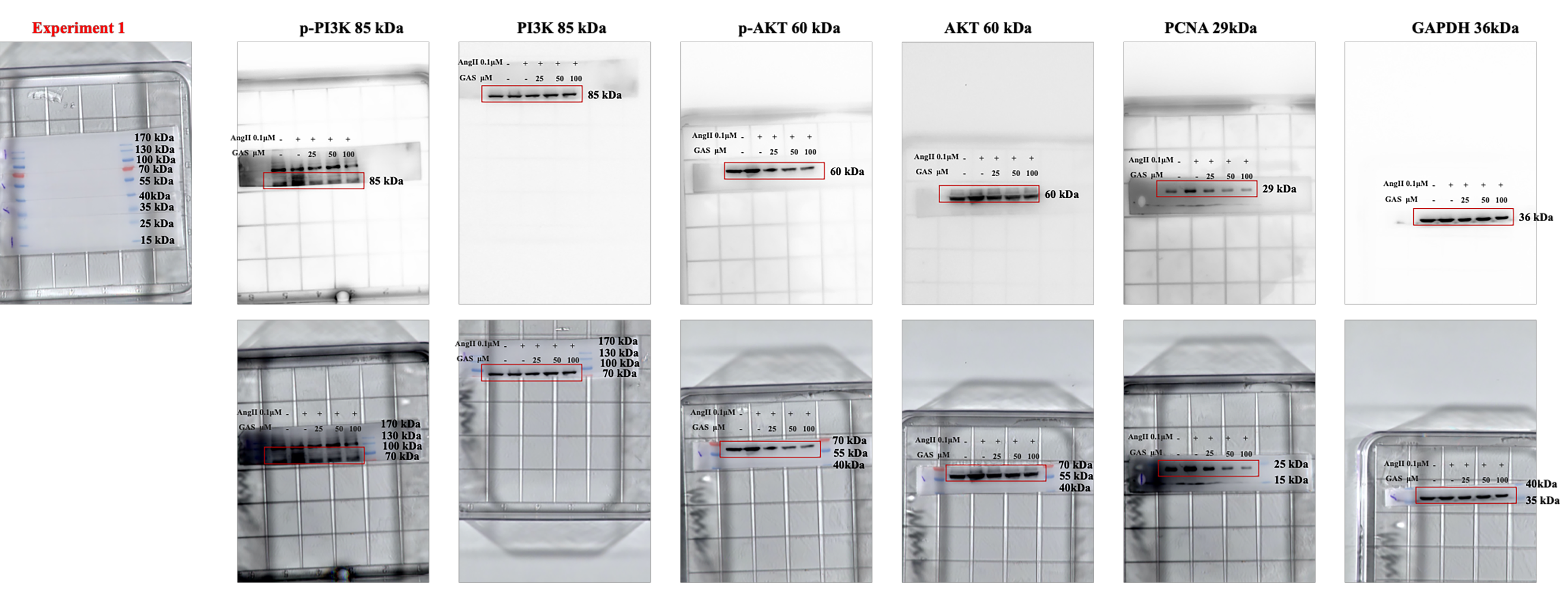

Supplement: Supplementary file 2 — Supplementary Figure S2. [file 41598_2023_39202_MOESM2_ESM.tif]

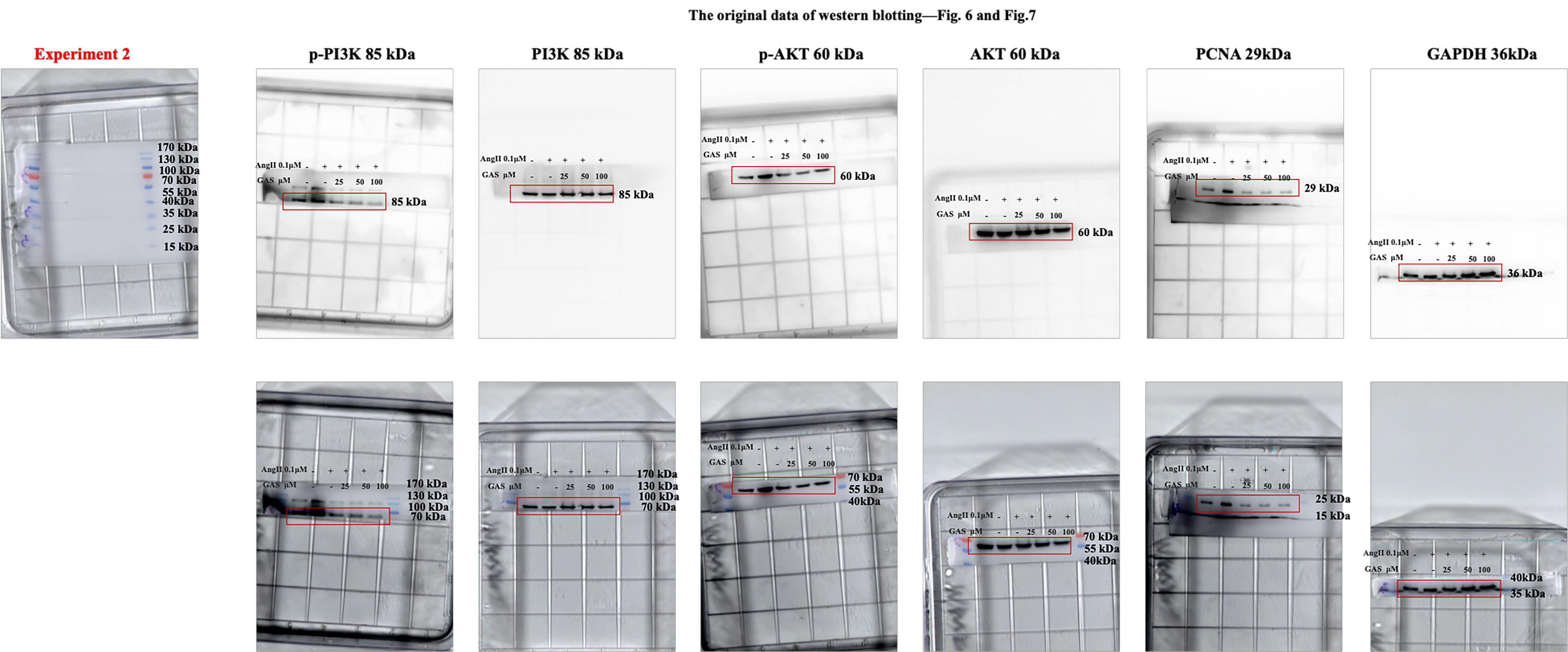

Supplement: Supplementary file 3 — Supplementary Figure S3. [file 41598_2023_39202_MOESM3_ESM.tif]

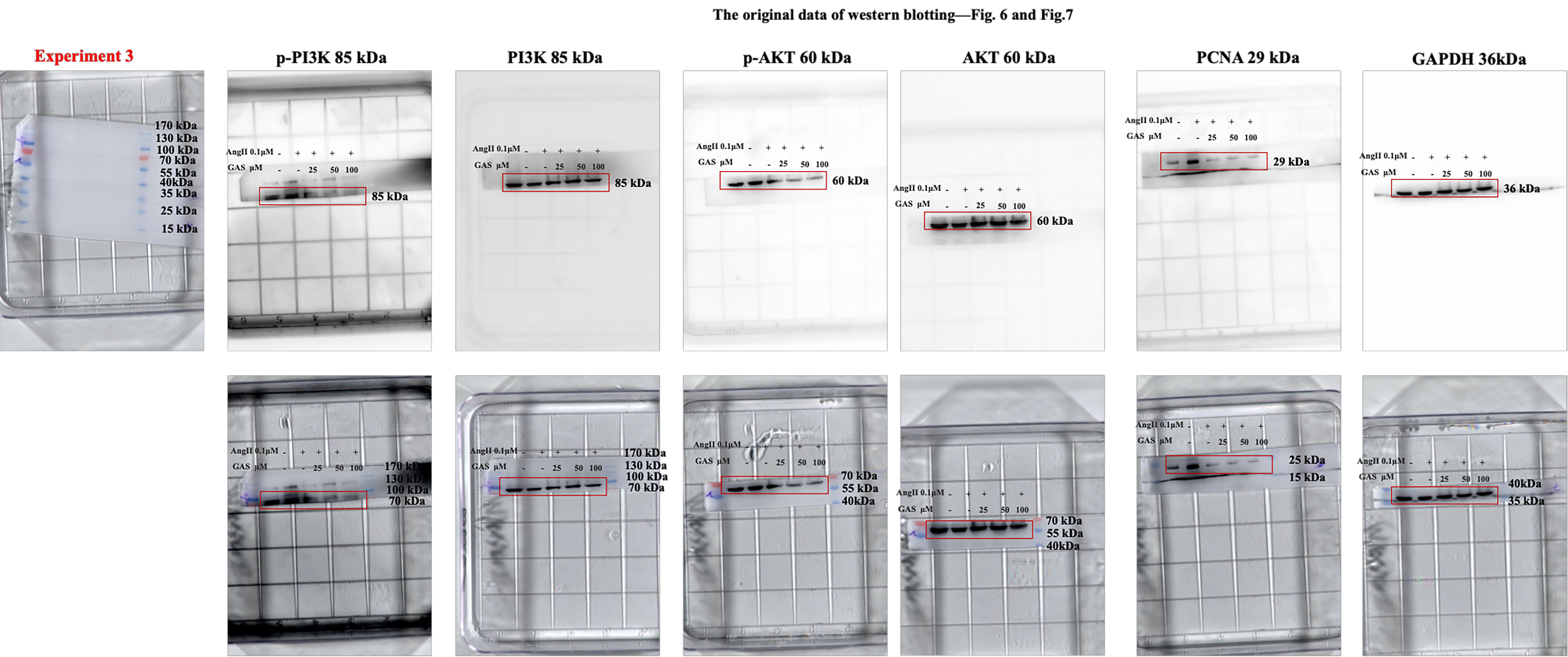

Supplement: Supplementary file 4 — Supplementary Figure S4. [file 41598_2023_39202_MOESM4_ESM.tif]
